# Dynamics of IgG antibody response against *Plasmodium* antigens among Nigerian infants and young children

**DOI:** 10.3389/fimmu.2023.1208822

**Published:** 2023-08-24

**Authors:** Colleen M. Leonard, Perpetua Uhomoibhi, Ado Abubakar, Abiodun Ogunniyi, Nwando Mba, Stacie M. Greby, McPaul I. Okoye, Nnaemeka C. Iriemenam, Chikwe Ihekweazu, Laura Steinhardt, Eric Rogier, NMS4 Technical Working Group

**Affiliations:** ^1^ Division of Parasitic Diseases and Malaria, Centers for Disease Control and Prevention, Atlanta, GA, United States; ^2^ Oak Ridge Institute for Science and Education, U.S. Department of Energy, Oak Ridge, TN, United States; ^3^ National Malaria Elimination Programme, Federal Ministry of Health, Abuja, Nigeria; ^4^ Institute of Human Virology (IHVN), Abuja, Nigeria; ^5^ Nigeria Centre for Disease Control (NCDC), Abuja, Nigeria; ^6^ Division of Global HIV and Tuberculosis, Center for Global Health, Centers for Disease Control and Prevention, Abuja, Nigeria

**Keywords:** malaria, antigens, maternal IgG, passive antibodies, humoral immunity

## Abstract

**Background:**

*Plasmodium falciparum* malaria is a leading cause of child mortality in Nigeria. Neonates are born with maternal antibodies from placental transfer which may protect against malaria infection in the first months of life. The IgG dynamics of the transition from passively transferred antimalarial antibodies to actively acquired IgG from natural exposure have not been well elucidated.

**Methods:**

Blood samples collected during a 2018 Nigeria nationwide HIV/AIDS household survey were available for 9,443 children under 5 years of age, with a subset of infants under 2 months of age having maternal samples available (n=41). Samples were assayed for the *P. falciparum* HRP2 antigen and anti-malarial IgG antibodies. LOESS regression examined the dynamics in IgG response in the first 5 years of life. Correlation with maternal IgG levels was assessed for mother/child pairs.

**Results:**

Consistent decreases were observed in median IgG levels against all *Plasmodium* spp. antigen targets for the first months of life. At a population level, *P. falciparum* apical membrane antigen-1 (AMA1) and merozoite surface protein-1 19kD (PfMSP1) IgG decreased during the first 12 months of life before reaching a nadir, whereas IgGs to other targets only declined for the first 4 months of life. Seropositivity showed a similar decline with the lowest seropositivity against AMA1 and PfMSP1 at 10-12 months, though remaining above 50% during the first 2 years of life in higher transmission areas. No protective association was observed between IgG positivity and *P. falciparum* infection in infants. Maternal antibody levels showed a strong positive correlation with infant antibody levels for all *P. falciparum* antigens from birth to 2 months of age, but this correlation was lost by 6 months of age.

**Discussion:**

Maternally transferred anti-malarial IgG antibodies rapidly decline during the first 6 months of life, with variations among specific antigens and malaria transmission intensity. From 3-23 months of age, there was a wide range in IgG levels for the blood-stage antigens indicating high individual variation in antibody production as children are infected with malaria. Non-*falciparum* species-specific antigens showed similar patterns in waning immunity and correlation with paired mother’s IgG levels compared to *P. falciparum* antigens.

## Introduction

Malaria is a leading cause of morbidity and mortality in sub-Saharan Africa, and in Nigeria, it is estimated that 14% of all under-five deaths are due to malaria (1). In countries with high levels of malaria transmission, infants are typically born with immunoglobulin G (IgG) antibodies against the endemic *Plasmodium* spp., which are transferred through the placenta while *in utero* (2). Additionally, there is evidence that anti-*Plasmodium* IgG can also be passively transferred through breast milk (3). Infants are partially protected against malaria infection and severe malaria for the first 3-6 months of life (4), and it has been well-documented that malaria infections among young infants (< 6 months old) are typically of low parasite density, asymptomatic, and clear spontaneously (5–7). Maternally-derived anti-*Plasmodium* IgG antibodies are thought to provide protection against this bloodborne pathogen during the first few months of life, though the mechanistic role(s) of these antibodies are not well elucidated for preventing malaria infection or febrile disease (8, 9). Besides passively-transferred IgG, other factors that may prevent malaria infection in infants include high levels of infant lactoferrin (which binds to iron) and secretory IgA (at high concentrations in breast milk) which have been found to inhibit *in vitro* growth of *P. falciparum* (10). Fetal hemoglobin (HbF) inhibits parasite development and provides another layer of protection for the first few months of life (6, 11). A study by Amaratunga, et al. suggests that HbF and IgG against *P. falciparum* PfEMP-1 cooperate to impair the cytoadherence of parasitized red blood cells in infants and thus substantially reduce the severity of *Plasmodium* infection (11). As HbF and maternal IgG levels decline, infants experience increased susceptibility to malaria infection and adverse outcomes. Depending on the level of malaria transmission in a setting, older infants (or children) have a higher cumulative risk of exposure to *Plasmodium* infection and would only be able to develop their own immune responses following such malaria parasite exposure; therefore, there may be a gap between the protection provided by neonatal factors together with maternal passive antibody transfer and the acquired response of the young person’s adaptive immune system.

Protection against malaria for the first year of life is dependent on the transmission intensity in the area and the month of infant birth (due to seasonal malaria exposure). Infants born in areas of higher transmission have been found to be generally protected for approximately 3 months (12), while in areas of low transmission, the risk of clinical malaria remains low for the first 6 months of life (13). Consistent with this association between transmission intensity and infants’ protection against malaria, it has been hypothesized that the apparent protection seen in young infants may merely result from limited exposure time to malaria due to their young age (14). Additionally, adults and older children are more often bitten by *Anopheles* mosquitoes compared to younger children and infants (15, 16). However, some studies have found that higher maternal antibody levels provide more extended protection against *P. falciparum* infection compared to infants with low maternal antibody levels, irrespective of transmission intensity (17).

This study reports on findings from IgG antibody and antigen assays from blood samples obtained from children and their mothers who were enrolled at their place of residence during the 2018 Nigeria HIV and AIDS Indicator Survey (NAIIS). In order to describe antimalarial IgG dynamics in early life in this Nigerian population, trends in antibody levels over the first 2 years of life and correlation between paired maternal and very young infant antibody levels are assessed. The antibody levels of very young infants (< 2 months old) were also compared between lower and higher *P. falciparum* transmission areas of Nigeria.

## Materials and methods

### Ethics statement

All participants provided informed consent before enrollment into the NAIIS survey. For children <10 years of age, the parent or guardian required written consent for biomarker testing and consent to future testing. Assent was also received from the children that were <10 years of age. Secondary laboratory testing for malaria biomarkers and laboratory data collection were approved by the National Health Research Ethics Committee of Nigeria (NHREC/01/01/2007) and were determined not to be involved in human research by the U.S. Centers for Disease Control and Prevention (CDC) Human Subjects office (project 0900f3eb819d4c63).

Ethical approval for secondary laboratory data collection of malaria biomarkers was approved for the Nigeria Multi-disease Serologic Surveillance using Stored Specimens (NMS4) project by the NHREC and U.S. CDC. All multiplex antigen detection and IgG detection assays were performed at the National Reference Laboratory (NRL) of the Nigeria Centre for Disease Control (NCDC) in Abuja. Multiplex antigen and IgG detection were attempted for all dried blood spot (DBS) specimens for children <15 years of age, and a subset of women of reproductive age (15-44 years). The data analysis shown in this article will focus on children under 5 years of age and paired mothers included in the women of reproductive age cohort.

### Study design

The NAIIS survey was a cross-sectional national household HIV survey conducted in Nigeria from July through December 2018. The Nigerian Federal Ministry of Health and the National Agency for the Control of AIDS led the national population-based NAIIS survey. Further details on the sampling design are available in the NAIIS report (18).

Among sampled households (N= 101,580), every fourth household was randomly selected for data and blood biomarker collection of children under 15 years of age. Laboratory staff collected 6 mL venous blood from children aged 2-14 years and 1 mL capillary blood (finger or heel stick) from children <2 years old. Whole blood samples were stored in cooler boxes and transported to satellite laboratories for processing into plasma and DBS within 24 hours of collection. Specimens were stored in -20°C freezers and transported to the central laboratory within a week where they were stored in -80°C freezers.

### Multiplex bead-based *Plasmodium* antigen detection assay

To rehydrate blood samples from DBS, a 6-mm punch (approximately 10 µL whole blood) was taken from each DBS sample and blood was eluted to a 1:40 concentration in blocking buffer (Buffer B: Phosphate Buffered Saline (PBS) pH 7.2, 0.5% Bovine Serum Albumin (BSA), 0.05% Tween 20, 0.02% sodium azide, 0.5% polyvinyl alcohol, 0.8% polyvinylpyrrolidone, and 3 µg/mL *Escherichia coli* extract) (19). Elutions were stored at 4°C until testing. As described previously (20), samples were assayed at the 1:40 whole blood dilution for *P. falciparum* histidine-rich protein 2 (HRP2). Assay plates were read on MAGPIX™ machines (Luminex Corporation, Austin, TX), and with a target of 50 beads/region, the median fluorescence intensity (MFI) was generated for each analyte. For each assay plate, the buffer background was subtracted for each target’s MFI signal to provide an MFI-bg assay signal for analysis. To determine the assay signal threshold for antigen positivity, a finite mixture model (FMM) approach was employed to estimate the ‘antigen negative’ population within these data (using data from specimens from all children <15 years of age from 36 states and FCT plus a sample of 9,191 women of reproductive age with completed antigen testing) and then establishing a cutoff using the mean + two standard deviations from this distribution (21).

### Multiplex bead-based IgG detection assay

The IgG-detecting multiplex bead assay (MBA) was performed as described previously (19). The four *Plasmodium* merozoite surface protein 1, 19kD (MSP1) antigens from *P. falciparum*, *P. malariae*, *P.* ovale, and *P. vivax* were utilized to assess IgG levels to different *Plasmodium* spp. Additionally, the following *P. falciparum* antigens were also included: apical membrane antigen-1 (AMA1), circumsporozoite protein (CSP), liver-stage antigen (LSA1), glutamate-rich protein R_0_ fragment (GLURP-R_0_), *Schistosoma japonicum* glutathione-*S*-transferase (GST) served as a control antigen (22). Further details for each antigen used in this multiplex IgG detection assay are included in **Supplementary Table 1**. From the DBS elution described above, an approximate serum dilution of 1:400 was used for the assay. Any DBS with GST MFI-bg reads above 500 were excluded from the analysis for quality assurance purposes to remove samples with potential non-specific IgG binding (23). To dichotomize seropositivity to each antigen, a 2-component FMM of the log_10_-transformed MFI-bg signal was fit for each antigen’s IgG response (22), and the positivity cutoff was determined by the lognormal mean of the first component (presumed seronegative) plus three standard deviations (21). Due to high levels of *P. falciparum* exposure and seropositivity, a 6-component FMM was created for PfMSP1 and AMA1 to best define the first, putative seronegative, component (24). Due to the transition between passively-transferred IgG in child serum, and actively acquired serum antibodies through natural exposure, different seropositivity cutoffs were generated for the population of children under 2, and the population of children from 2 to 4 years of age.

### Statistical methods

FMM plots were generated in SAS version 9.4 (SAS Institute, Cary, NC). All remaining analyses were conducted in R version 4.1.1 (R Foundation for Statistical Computing, Vienna, Austria). Locally estimated scatterplot smoothing (LOESS) regression was used to show the proportion of children seropositive by age in months up until 4 years of age. Age in months was only available for children under 2 years, and seropositivity was plotted at the midpoint of the age group above 2 years (e.g., 30 months for 2 years of age). We also analyzed the proportion of children seropositive by age group stratified by relative lower and higher transmission areas of Nigeria. As an objective measure of *P. falciparum* transmission in the population through antimalarial antibody carriage, the definition for a higher transmission area of Nigeria was inclusive of any state at or above the median of seropositivity to PfMSP1 among the children < 5 years of age. As a proxy for previous *P. falciparum* exposure, this metric was utilized only to classify Nigerian states into relatively higher or lower transmission of *P. falciparum.* Demographics and malaria positivity estimates were adjusted considering the complex sample design using the R survey package (25). Mother and child IgG levels were assessed for available pairs for several age groups (children under 1 month, 1 month, 2 months, 6 months, etc.) fitting a linear regression line on a scatterplot. Kendall’s rank correlation coefficient (τ) was used to determine the correlation between paired maternal and very young infants’ IgG levels. Any IgG MFI-bg levels < 1.0 were assigned a value of 1.0 to be included in the analysis when log-transforming the MFI-bg values.

## Results

A total of 9,487 DBS samples from children under 60 months of age were available for testing by the multiplex assay. Of these, 44 (0.5%) had a GST assay signal above 500 and were excluded from the analysis for quality assurance (23), leaving a total of 9,443 samples available for analysis. **Table 1** presents the demographic characteristics of the children for whom samples are included in this analysis. Compared with the other Nigerian zones, a higher percentage of children were from the North West Zone (27.9%), though the number of children from any one zone was over 1,000 (range: 1,241-2,630). The number of children with serology data available for analysis by state in Nigeria is shown in **Supplemental Table 2** (median: 220; range: 74-563). Representation by sex was even: 50.9% male vs. 49.1% female.

**Table 1:** Demographic characteristics of the study population with serological data collected: Nigeria, 2018.

	Children under 24 months of age	Children under 60 months of age
**Total participants (n)**	2,705	9,443
**Age in months, mean (range)**	11.8 (0-23)	34.2 (0-54)
Geopolitical Zone n (%)		
North East	388 (14.3)	1,490 (15.8)
North Central	387 (14.3)	1,411 (14.9)
North West	713 (26.4)	2,630 (27.9)
South West	408 (15.1)	1,315 (13.9)
South East	352 (13.0)	1,241 (13.1)
South-South	457 (16.9)	1,356 (14.4)
Sex, n (%)		
Male	1,329 (50.9)	4,809 (50.9)
Female	1,376 (49.1)	4,634 (49.1)

**Figure 1:**
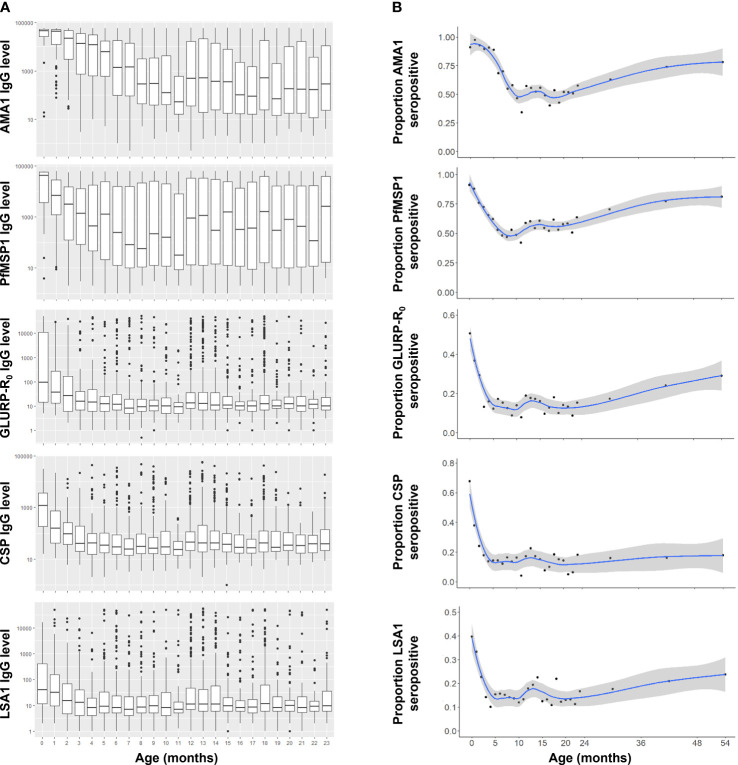
Anti-*Plasmodium falciparum* antibody dynamics in Nigerian children under the age of five years. **(A)** IgG levels for children 0-23 months of age. Boxes display interquartile range (IQR) and whiskers extend 1.5x IQR above and below. Markers indicate observations outside of 1.5x IQR. Medians are displayed by horizontal lines. **(B)** IgG seropositivity by age to *P. falciparum* antigens for 0-59 month-old-children with smoothed LOESS regression curve (solid line) and 95% confidence intervals (shading).

The youngest children (less than 2 months of age) had the highest median IgG levels to all five *P. falciparum* antigens utilized in this panel (**Figure 1A**), as well as the three non-falciparum MSP1 antigens (**Supplementary Figure 1A**). As infants aged in this population, decreases were observed in median IgG levels, with the longest monotonic decreases in median IgG levels observed for 11 months (for AMA1) and the shortest monotonic decreases for the non-falciparum MSP1 antigens (all 3 months or less). Upon reaching the lowest point for IgG levels to any *Plasmodium* antigen, population median IgG levels up to 24 months of age were highly variable for the AMA1 and PfMSP1 antigens but variation was generally low for all other IgG targets (**Figure 1A**). Between 0 and 6 months of age, significant reductions in IgG levels were observed for all eight IgG targets, with the greatest reduction in CSP IgG (by 52.0%) and the smallest reduction in PvMSP1 IgG (by 15.1%) (**Table 2**). All *P. falciparum* antigens saw a minimum 32.2% decrease in the population IgG levels for infants as they aged from 0 to 6 months.

**Table 2:** Loss of anti-*Plasmodium* IgG antibodies between <1 month and 6 months of life.

Antigen	Seropositivity of neonates <1 month (%, 95% CI)	Monotonic decrease in median IgG (# of months)	Log-transformed median IgG level at <1 month old	Log-transformed median IgG level at 6 months old	Percent change: <1 vs 6 months	p-value*
*P. falciparum* AMA1	90.9 (69.4, 98.4)	11	10.7	7.21	-32.6%	<0.0001
*P. falciparum* MSP1	90.9 (69.4, 98.4)	4	10.6	5.50	-48.1%	<0.0001
*P. falciparum* CSP	68.2 (45.1, 85.3)	7	7.08	3.40	-52.0%	<0.0001
*P. falciparum* LSA1	45.5 (25.1, 67.3)	4	3.67	2.08	-43.3%	0.002
*P. falciparum* GLURP-R_0_	50.0 (30.7, 69.3)	7	4.55	2.44	-46.4%	<0.001
*P. malariae* MSP1	54.5 (32.7, 74.9)	3	5.63	2.89	-48.7%	<0.0001
*P. ovale* MSP1	13.6 (3.6, 36.0)	2	3.38	2.56	-24.3%	0.002
*P. vivax* MSP1	9.1 (1.6, 30.6)	2	2.71	2.30	-15.1%	0.036

*p-values calculated for IgG levels of <1 month versus 6-month-old study populations using the two-sample Wilcoxon rank-sum test.

For all *Plasmodium* antigens, IgG seropositivity among <1 month-old neonates (essentially at birth) ranged from 9.1% to 90.9% (**Table 2**). *P. falciparum* IgG targets reached a seropositivity low point between 10-18 months of age, but then continually increased up to the oldest ages assessed here: 59 months (**Figure 1B**). The low point for seropositivity for the *P. falciparum* AMA1 and PfMSP1 targets remained steady at approximately 40-60% up to 24 months of age, but by five years of age, 78.1% and 81.1% of the children had a positive IgG response to AMA1 and PfMSP1, respectively. At the population level, increases in seropositivity were also observed for the GLURP-R_0_, CSP, and LSA1 IgG targets as children approached the age of 5 years, though not at the same rate as the AMA1 and PfMSP1 antigens. Between 24 months and 5 years of age, steady increases in seropositivity were also seen for the PmMSP1, PoMSP1, and PvMSP1 antigens (**Supplemental Figure 1B**).

**Figure 2:**
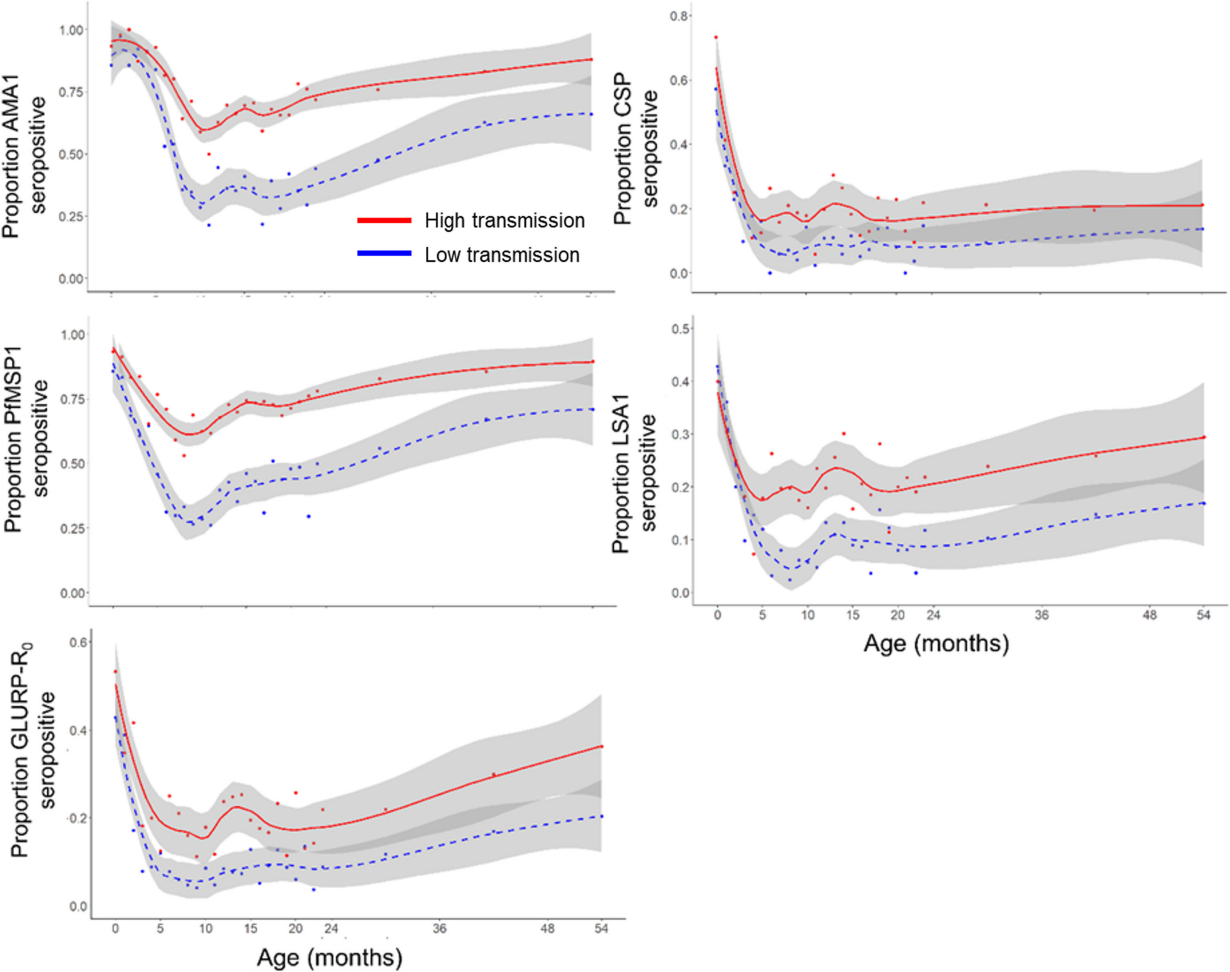
Seropositivity to *Plasmodium falciparum* antigens by relative transmission intensity. Smoothed LOESS regression curves indicate estimates by lower transmission (blue hashed line) and higher transmission (red solid line) settings in Nigeria. Grey shading around LOESS curves indicates a 95% confidence interval.

When comparing populations of children living in areas of relatively different *P. falciparum* transmission intensity (as defined in Methods, and displayed in **Supplemental Table 2** and **Supplemental Figure 2**), the proportion of neonates (<1 month old) seropositive to different *Plasmodium* targets between higher and lower transmission areas was similar among all targets (**Figure 2**) with no statistically significant differences found in IgG levels to any of the *P. falciparum* targets (**Supplementary Table 3**). However, as children in the population aged from 0 to 10 months, sharper decreases were noted in loss of IgG seropositivity (seroreversion) in populations of children residing in relatively lower transmission areas versus the higher transmission group, and this trend was most pronounced for the AMA1, PfMSP1, and LSA1 antigens. For all *P. falciparum* IgG targets, the LOESS curves for child seropositivity rates were separated with children in the higher transmission settings consistently showing higher seropositivity. As stratified by the *P. falciparum* transmission setting, seropositivity to the non-falciparum targets was generally higher in higher *P. falciparum* areas, but was more variable due to overall lower seropositivity to non-falciparum antigens (**Supplemental Figure 3**).

HRP2 antigenemia during the first 2 months of life could be a factor of congenital malaria infection (26), placental malaria *in utero* (27), or *P. falciparum* infection acquired after birth. In this Nigerian population, young children were found to be more likely to carry HRP2 antigen as they aged. HRP2 positivity among all 0- to 5-month-old children was 18.3% which increased to 22.8% in 6- to 11-month-olds, 29.9% in 12- to 17-month-olds, and 27.0% in 18- to 23-month-olds. In comparing HRP2 antigenemia among infants less than 6 months of age to the paired maternal samples, the association for frequency of antigenemia was not found to be significant (Fisher’s exact p-value = 0.15, **Supplementary Table 4**). Differences in IgG seropositivity among young children were assessed by HRP2 antigenemia status. Though HRP2-positive infants typically had higher seropositivity rates for *Plasmodium* antigens versus HRP2-negative infants, no substantial differences in seropositivity were observed for all five *P. falciparum* antigens and the three non-falciparum MSP1 antigens (**Supplementary Table 5**).

**Figure 3:**
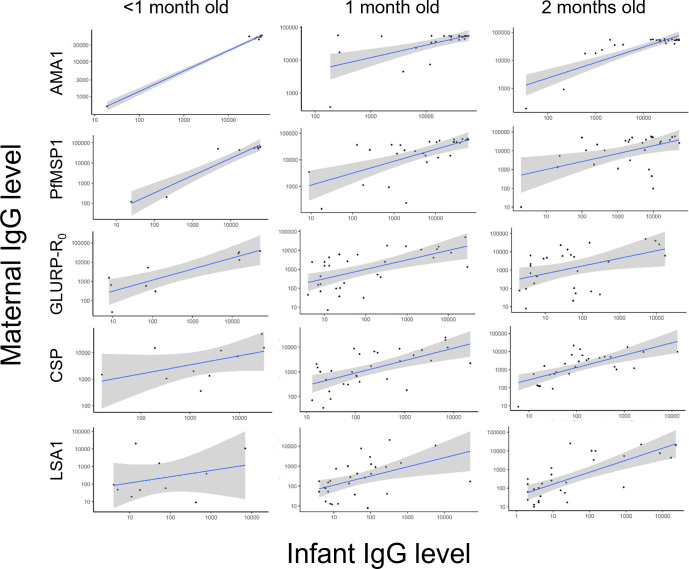
Scatterplots of paired maternal and very young infants’ anti-malarial IgG levels against *P. falciparum* antigens. A solid line shows linear regression fitting for the data with shading indicating a 95% confidence interval.

A total of 41 pairs of DBS samples had IgG results available for infants less than 2 months old and their mothers. Overall, there was a strong correlation between these infants’ and their mothers’ IgG levels for all *Plasmodium* antigens (median Kendall’s τ= 0.45) (**Table 3**, **Figure 3**), with the highest correlation for PfMSP1 (τ= 0.65) and lowest for LSA1 and PvMSP1 (τ= 0.29). Except for PmMSP1 IgG levels, median IgG levels to all other *Plasmodium* antigens were consistently higher in mothers compared to their corresponding infants (**Supplementary Figure 4**). As children age, the correlation between infant and mothers’ IgG levels is essentially lost after approximately 6 months of age as shown in **Supplementary Figure 5**. HRP2 antigenemia among children under 2 years of age was compared by mother’s IgG levels as stratified by tertiles and categorized by different child age groups (**Supplementary Table 6**, **Supplementary Figure 6**). For all *P. falciparum* IgG targets assessed, mothers with higher IgG levels generally had a higher proportion of children with HRP2 antigenemia compared with mothers with lower IgG levels to the *P. falciparum* targets.

**Table 3:** Correlation of IgG levels between infants less than 2 months of age and their mothers.

	Log IgG level, mothers Median (IQR)	Log IgG level, infants Median (IQR)	τ (p-value)	Linear regression slope (R^2^, p-value)
*P. falciparum* AMA1	10.9 (10.7- 10.9)	10.6 (9.6- 10.8)	0.42 (< 0.001)	0.58 (< 0.001)
*P. falciparum* MSP1	10.7 (9.6- 10.9)	9.4 (7.3- 10.7)	0.65 (< 0.001)	0.56 (< 0.001)
*P. falciparum* CSP	7.4 (6.2- 8.9)	5.5 (4.0- 7.7)	0.44 (< 0.001)	0.41 (< 0.001)
*P. falciparum* GLURP-R0	7.4 (5.3- 8.9)	4.2 (2.3- 7.7)	0.50 (< 0.001)	0.47 (< 0.001)
*P. falciparum* LSA1	5.1 (3.9- 6.8)	3.3 (2.1- 4.6)	0.29 (<0.01)	0.19 (<0.01)
*P. malariae* MSP1	4.7 (3.7- 7.0)	5.4 (3.3- 8.4)	0.46 (< 0.001)	0.36 (< 0.001)
*P. ovale* MSP1	4.2 (3.1- 6.1)	3.1 (2.3- 4.4)	0.50 (< 0.001)	0.54 (< 0.001)
*P. vivax* MSP1	4.1 (3.1- 5.7)	2.5 (2.1- 3.4)	0.29 (< 0.01)	0.40 (< 0.001)

IQR, interquartile range; τ = Kendall’s rank correlation tau.

## Discussion

Neonates are born with a variety of different serum and immunological factors which offer a level of protection against the establishment of *Plasmodium* spp. infection and development of severe malaria. One of the most abundant factors in terms of serum concentration is IgG antibodies which are transferred across the fetal placenta *in utero* (8, 28, 29). For neonates and young infants across Nigeria, this current study found IgG levels against *P. falciparum* sporozoite, liver-stage, and blood-stage antigens were all very high during the first 3 months of life and quickly waned as infants aged through their first year of life. Median IgG levels against the AMA1 and PfMSP1 antigens decreased to their lowest level in infants around 11 months of age but reached a low point around 5-7 months of age for CSP, GLURP-R_0_, and LSA1. IgG levels for these latter three targets remained low throughout the first 2 years of life, but high variability in AMA1 and PfMSP1 antigens as children aged showed *P. falciparum* exposure and adaptive immune responses to these immunogenic antigens. Maternal transfer of non-falciparum IgG was also noted in this neonatal study population (24) and showed similar temporal dynamics to the *P. falciparum* IgG targets. Similar trends of waning maternally-derived IgG levels have been found in previous studies, with antimalarial IgG levels decreasing to their lowest between 6-9 months of age in other high-transmission settings and the lowest proportion of seropositive infants was seen at approximately 10 months for AMA1 and PfMSP1 (30, 31). These previous findings, in conjunction with the results presented here, suggest that even in very high transmission and holoendemic settings, infants have lost all passively-transferred maternal IgG by their first birthday.

Several studies have shown that maternal antibodies in cord blood provide protection against severe malaria in infants (32, 33). One longitudinal study found that infants with high anti-PfSEA-1 antibody levels in cord blood had 51% fewer cases of severe malaria compared to those with lower antibody levels over the 12-month follow-up period (33). Some previous studies have observed a protective effect of maternal antibodies against any malaria infections in infants while others have not (8, 34). Branch and colleagues found that infants with high IgG and IgM levels to MSP-1-19kD antigen experienced a longer time to first infection (113 vs. 69 days) in a Kenyan cohort (35). Importantly, all participants from this current Nigerian survey were enrolled in their households, so any *P. falciparum* infections identified here would most likely be described as low-density and asymptomatic, or at least not exhibiting treatment-seeking behavior at the time of sampling. This current study utilized a relatively small panel of five *P. falciparum* antigen targets for IgG detection and was only able to identify the presence of *P. falciparum* by HRP2 antigenemia. Taking these points into consideration, higher levels of maternal IgG to these *P. falciparum* antigens did not have a protective effect in reducing rates of *P. falciparum* in the corresponding children. In fact, mothers with higher IgG levels consistently had children with higher rates of HRP2 antigenemia. This association is most likely due to the fact that the higher IgG levels of mothers are a reliable proxy for sustained *P. falciparum* transmission in their area of residence, so their children residing in the same area are also subjected to these higher levels of *P. falciparum* exposure (30).

It is documented that it takes many occurrences of natural exposure to *P. falciparum* to develop an immune response capable of preventing clinical malaria (36). Even in higher transmission settings, resistance through adaptive immune responses to the most severe forms of malaria is obtained through multiple natural exposures over 5 years, and resistance to any clinical malaria begins to be attained by late childhood or early adolescence (37). The findings presented here show that from 3 to 23 months of age, there was a wide interquartile range in IgG levels against the blood-stage antigens. This likely indicates that there are many factors that influence antibody levels in the first 2 years of life, including the amount of malaria exposure, behavioral factors, and protective measures that are utilized. Passively transferred maternal anti-malarial IgG was consistently highest within the first 2 months of life. Along with IgG to other pathogen-specific antigens, IgG antibodies against malaria parasites are transferred *via* the placenta during fetal development (2). In this study, it was observed that mother and infant IgG levels are highly correlated for infants less than 2 months old, with strong mother/child correlation in the quantitative levels of AMA1, PfMSP1, CSP, and GLURP-R_0_ antigens. A previous study in Kenya testing placental cord blood and found that 97% of samples were positive for AMA1 IgG antibodies and 87% tested positive for CSP IgG antibodies (30). Other studies have detected maternal IgG antibodies against *P. falciparum* AMA1, CSP, GLURP-R_0_, and MSP1-19 antigens in cord blood (29, 32, 38, 39). This current study found neonatal antimalarial IgG levels were generally lower compared to their mothers’ levels, and this is consistent with other studies conducted in different *P. falciparum* transmission settings (8, 28, 40, 41).

When assessing children living in the lower malaria transmission areas of Nigeria, a more rapid decline in seropositivity was observed for the *P. falciparum* antigens compared to the children in higher transmission areas. This is most likely due to lower *P. falciparum* exposure in lower transmission areas compared to the higher transmission settings. Between 5-24 months of age, most children in lower transmission areas were seronegative to the AMA1 and PfMSP1 antigens, while seropositivity never fell below 50% for the children in higher transmission areas. One study that followed children in Kenya during a year of decreasing malaria transmission identified that the average antibody levels against AMA1 and MSP2 significantly declined in all age groups between the start and end of the year (42).

This study is subject to several limitations. Primarily, the cross-sectional design does not allow for longitudinal assessment of an individual child’s IgG levels over time, so the assumption by the analyses is that infants sampled at each age category offer a proxy for children as they age from month to month in Nigeria. Though nearly 10,000 samples were available from children less than 5 years of age in this study, an overall limited number of infant samples paired with maternal samples were available for assessing mother/child correlation (n= 66 for HRP2 antigenemia among children under 6 months and n= 41 for IgG data among children under 2 months). Additionally, in assessing HRP2 antigenemia as a measure of active infection, recent clinical history was unavailable for these persons, and HRP2 transfer across the placental barrier is poorly understood. Only eight *Plasmodium* antigens were utilized for IgG capture, though numerous *Plasmodium* antigens have been identified to date. Functional assays were not performed, nor was sub-typing of IgG antibodies, so total IgG binding to antigens was the only antibody data collected in this study. No data on symptomatic status nor clinical history (previous or post-survey) for these children was collected, so the assessment of *P. falciparum* infection was only considering if the child had evidence of infection at the time of sampling, and protection from clinical disease was unable to be ascertained. Infection or exposure to many other infectious agents endemic to Nigeria was not ascertained in this study, so potential IgG cross-reactivity could not be assessed here.

Understanding the transition from passively transferred maternal antibodies present in infant serum to the active adaptive immune responses of the host can assist in determining the risk of severe disease for infants in different malaria transmission settings. This work additionally found that antibodies to non-falciparum species-specific antigens showed similar patterns in waning immunity and the correlation with paired mother’s IgG levels was similar to *P. falciparum* antigens. Further studies should evaluate both maternal and neonatal factors associated with protective immunity in infants.

## Data availability statement

The original contributions presented in the study are included in the article/**Supplementary Material**, further inquiries can be directed to the corresponding author.

## Ethics statement

The studies involving human participants were reviewed and approved by National Health Research Ethics Committee of Nigeria. Written informed consent to participate in this study was provided by the participants’ legal guardian/next of kin.

## Author contributions

Project coordination: PU, AA, AO, NM, SG, MO, NI, CI, LS, and ER; Data collection: AA and ER; Formal analysis: CL; Manuscript drafting: CL and ER. All authors contributed to the article and approved the submitted version.

## Members of NMS4 technical working group

Oluwaseun O. Akinmulero, Andrew Thomas, Ayuba Dawurung, Mudiaga Eseikpe, Abubakar Bichi Iliyasu, Nengi Israel, Erasogie Evbuomwan, Chimaoge Achugbu, Mary Okoli, Nnaemeka Ndodo.
