# Supplementary material for: Dynamics of IgG antibody response against Plasmodium antigens among Nigerian infants and young children
Source: Front Immunol. 2023 Aug 24;14:1208822. doi: 10.3389/fimmu.2023.1208822 (PMC10484571; doi:10.3389/fimmu.2023.1208822)
Supplement: Supplementary file 1 [file DataSheet_1.pdf]

Supplementary Material

Dynamics of IgG antibody response against *Plasmodium* antigens among Nigerian infants and young children

Supplementary Table 1. Antigens utilized for IgG capture or assay control in this study.

Microbe Species	Antigen	Alias	Infection stage expression
Plasmodium falciparum	merozoite surface protein 1, 19kD fragment	PfMSP1	Blood stage
Plasmodium falciparum	apical membrane antigen, N terminal region	AMA1	Blood stage
Plasmodium falciparum	Gutamate-rich protein, R_0 fragment	GLURP-R_0	Blood stage
Plasmodium falciparum	circumsporozoite protein, (NANP)x5	CSP	Sporozoite stage
Plasmodium falciparum	liver stage antigen 1, Pl1043 epitope	LSA1	Liver stage
Plasmodium malariae	merozoite surface protein 1, 19kD fragment	PmMSP1	Blood stage
Plasmodium ovale	merozoite surface protein 1, 19kD fragment	PoMSP1	Blood stage
Plasmodium vivax	merozoite surface protein 1, 19kD fragment	PvMSP1	Blood stage
Schistosoma japonicum	Glutathione-*S*-transferase	GST	NA

Supplementary Table 2. Children with serology data available and *Plasmodium falciparum* transmission intensity by state: Nigeria, 2018.

STATE	Number of samples for analysis	Transmission intensity*
Abia	202	Low
Adamawa	215	High
Akwa Ibom	271	High
Anambra	286	Low
Bauchi	421	High
Bayelsa	141	Low
Benue	311	Low
Borno	163	Low
Cross River	188	High
Delta	258	Low
Ebonyi	208	High
Edo	210	Low
Ekiti	110	High
Enugu	206	High
Federal Capital Territory	74	Low
Gombe	267	High
Imo	339	Low
Jigawa	427	High
Kaduna	563	High
Kano	456	Low
Katsina	486	High
Kebbi	290	High
Kogi	136	Low
Kwara	138	High
Lagos	459	Low
Nasarawa	136	Low
Niger	396	High
Ogun	175	Low
Ondo	144	Low
Osun	119	High
Oyo	308	Low
Plateau	220	Low
Rivers	288	Low
Sokoto	282	High
Taraba	232	High
Yobe	192	High

Zamfara	126	High

***** For the purposes of this study, state at or above the median of seropositivity to PfMSP1 among children <5 years was considered a high transmission area

Supplementary Table 3. Seropositivity and loss of anti-*Plasmodium falciparum* IgG antibodies during the first 6 months of life by *P. falciparum* transmission setting.

**Antigen and *P. falciparum* transmission setting**	**Seropositivity of neonates <1 month (%, 95% CI)**	**Log-transformed IgG level at <1 month old**	**p-value <1 month old***	**Log-transformed IgG level at 6 months of age**	**p-value 6 month old***	**Percent change: <1 vs 6 months**
**AMA1**						
High	93.3 (66.0, 99.7)	10.7	0.63	8.62	<0.001	-19.4%
Low	85.7 (42.0, 99.2)	10.8		5.29		-51.0%
**PfMSP1**						
High	93.3 (66.0, 99.7)	10.80	0.39	8.32	<0.001	-23.0%
Low	85.7 (42.0, 99.2)	9.74		3.07		-68.5%
**CSP**						
High	73.3 (44.8, 91.1)	7.07	0.78	3.47	0.17	-50.9%
Low	57.1 (20.2, 88.2)	7.66		3.38		-55.9%
**LSA1**						
High	46.7 (22.3, 72.6)	3.97	0.67	2.56	<0.001	-35.5%
Low	42.9 (11.8, 79.8)	3.30		1.79		-45.8%
**GLURP-R_0**						
High	53.3 (27.4, 77.7)	4.76	0.53	2.67	0.01	-43.9%
Low	42.9 (11.8, 79.8)	4.74		2.20		-19.7%

*p-values calculated for difference in IgG between relatively higher and lower transmission settings using a pairwise Wilcoxon rank-sum test

Supplementary Table 4. Comparison of mother vs. children *Plasmodium falciparum* HRP2 antigenemia among infants under 6 months of age.

		Mother	
		**HRP2+**	**HRP2-**
**Child**	**HRP2+**	3	5
	**HRP2-**	9	49

Supplementary Table 5. IgG seropositivity by *Plasmodium falciparum* HRP2 antigenemia among infants under 2 months of age.

Antibody Target	Overall percent seropositive	HRP2 antigen positive (n=14)	HRP2 antigen negative (n= 90)	p-value*
P. falciparum AMA1	96.2%	92.9%	96.7%	0.49
P. falciparum MSP1	92.3%	100.0%	91.1%	0.23
P. falciparum CSP	46.2%	50.0%	45.7%	0.79
P. falciparum GLURP-R0	41.4%	35.4%	42.3%	0.59
P. falciparum LSA1	36.7%	50.2%	34.5%	0.29
P. malariae MSP1	47.3%	57.3%	45.7%	0.44
P. ovale MSP1	13.5%	14.2%	13.4%	0.94
P. vivax MSP1	4.8%	14.2%	3.3%	0.07

*p-values calculated using Pearson's chi-square with Rao and Scott adjustment.

Supplementary Table 6. ***Plasmodium falciparum*** **HRP2 antigenemia among children under 2 years stratified by maternal IgG tertiles and categorized by child's age.**

Maternal Tertile	0-5 months		6-11 months		12- 17 months		18- 23 months	
	HRP2+ n(%)	*HRP2- n(%)*	*HRP2+ n(%)*	*HRP2- n(%)*	*HRP2+ n(%)*	*HRP2- n(%)*	*HRP2+ n(%)*	*HRP2- n(%)*
**Lowest tertile (AMA1)**	8 (15.5)	45 (84.9)	17 (20)	68 (80)	21 (17.6)	98 (82.4)	14 (20.3)	55 (79.7)
**Middle tertile (AMA1)**	16 (26.7)	44 (73.3)	21 (23.6)	68 (76.4)	33 (32.0)	70 (68.0)	25 (34.2)	48 (65.8)
**Highest tertile (AMA1)**	14 (20)	56 (80)	25 (29.8)	59 (70.2)	56 (44.4)	70 (55.6)	24 (42.9)	32 (57.1)
**Lowest tertile (PfMSP1)**	13 (21.3)	48 (78.7)	15 (16.0)	79 (84.0)	25 (22.9)	84 (77.1)	12 (19.4)	50 (80.6)
**Middle tertile (PfMSP1)**	9 (14.8)	52 (85.2)	21 (25.6)	61 (74.4)	42 (35.9)	75 (64.1)	22 (33.3)	44 (66.7)
**Highest tertile (PfMSP1)**	16 (26.2)	45 (73.8)	27 (32.9)	55 (67.1)	43 (35.0)	80 (65.0)	29 (41.4)	41 (58.6)
**Lowest tertile (GLURP-R0)**	12 (21.8)	43 (78.2)	12 (14.6)	70 (85.4)	24 (20.2)	95 (79.8)	18 (25.4)	53 (74.6)
**Middle tertile (GLURP-R0)**	13 (16.0)	68 (84.0)	21 (26.6)	58 (73.4)	31 (29.2)	75 (70.8)	15 (25.0)	45 (75.0)
**Highest tertile (GLURP-R0)**	13 (27.7)	34 (72.3)	30 (30.9)	67 (69.1)	55 (44.0)	70 (56.0)	30 (44.8)	37 (55.2)
**Lowest tertile (CSP)**	6 (10.2)	53 (89.8)	10 (12.1)	73 (88.0)	25 (20.5)	97 (79.5)	14 (22.2)	49 (77.8)
**Middle tertile (CSP)**	10 (16.9)	49 (83.1)	18 (20.2)	71 (79.8)	32 (28.6)	80 (71.4)	20 (30.3)	46 (69.7)
**Highest tertile (CSP)**	22 (33.8)	43 (66.2)	35 (40.7)	51 (59.3)	53 (45.7)	63 (54.3)	29 (42.0)	40 (58.0)
**Lowest tertile (LSA1)**	14 (20.0)	56 (80.0)	9 (12.5)	63 (87.5)	32 (25.6)	93 (74.4)	9 (15.5)	49 (84.5)
**Middle tertile (LSA1)**	8 (14.0)	49 (86.0)	29 (29.0)	71 (71.0)	42 (37.5)	70 (62.5)	23 (39.0)	36 (61.0)
**Highest tertile (LSA1)**	16 (28.6)	40 (71.4)	25 (29.1)	61 (70.9)	36 (31.9)	77 (68.1)	31 (38.3)	50 (61.7)

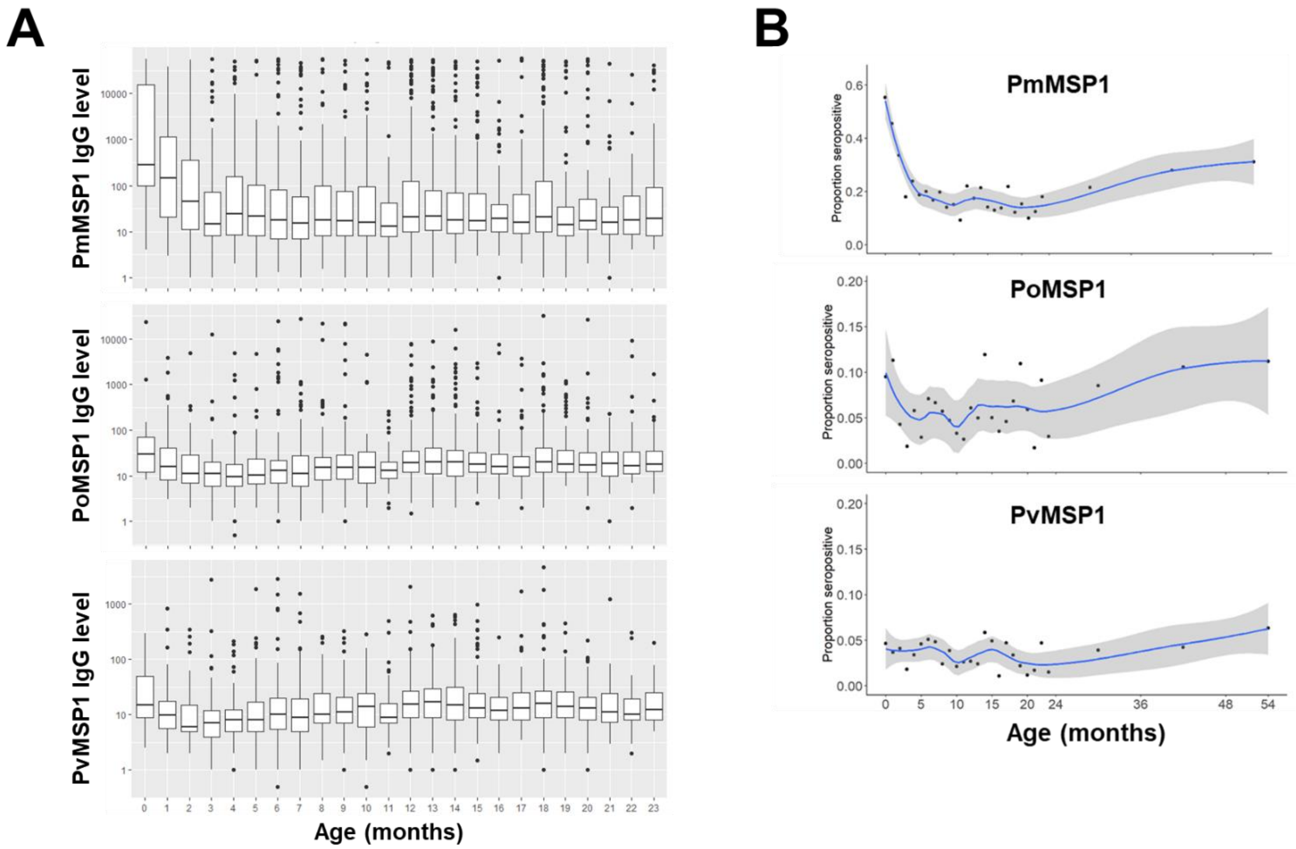

Supplementary Figure 1. IgG levels and seropositivity to *Plasmodium malariae*, *P. ovale*, and *P. vivax* MSP1 antigens in children: Nigeria, 2018. (A) IgG levels for children 0-23 months of age. Boxes display interquartile range (IQR) and whiskers extend 1.5x IQR above and below. Markers indicate observations outside of 1.5x IQR. Medians displayed by horizontal line. (B) IgG seropositivity by age to *P. falciparum* antigens for 0-59 month-old-children with smoothed LOESS regression curve (solid line) and 95% confidence intervals (shading).

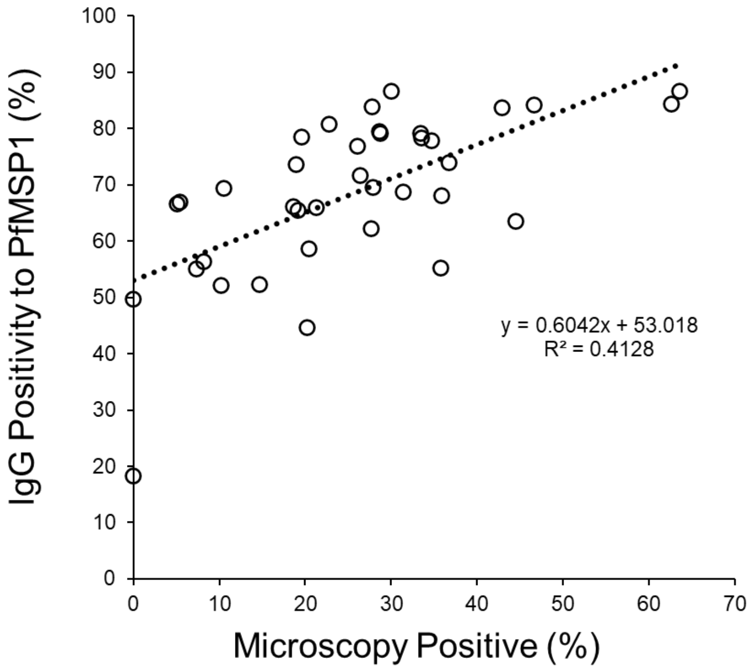

Supplementary Figure 2. State-level comparison of active *P. falciparum* infection (by microscopy) to seropositivity to PfMSP1 antigen. Scatterplot shows the percentage of children under 5 years microscopy positive for *P. falciparum* from previous cross-sectional survey compared with percent of children under 5 years from the current survey seropositive to the PfMSP1 antigen. Each marker in plot represents a state in Nigeria, and hashes line shows linear regression fitting with inset displaying regression estimates. State-level microscopy data obtained from the 2015 Nigeria Malaria Indicator survey (dhsprogram.com/pubs/pdf/MIS20/MIS20.pdf).

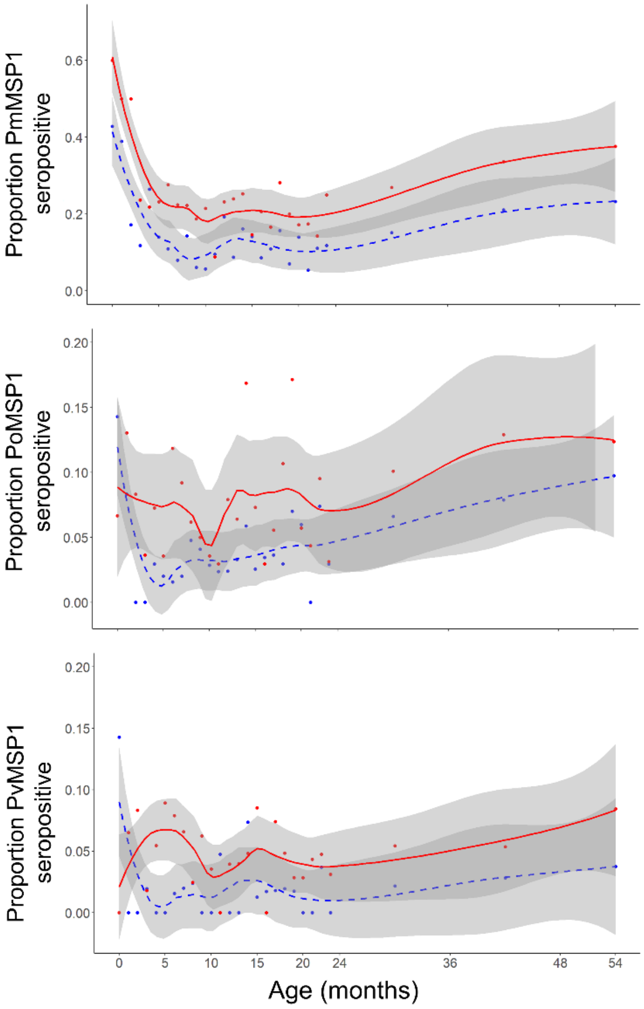

Supplementary Figure 3. Locally-smoothed seropositivity to non-*falciparum* antigens by *Plasmodium falciparum* transmission intensity. LOESS regression was used to estimate the smoothed regression curves with blue hashed lines indicating lower transmission areas and red solid lines higher transmission area. Grey shading around LOESS curves indicates the 95% confidence interval.

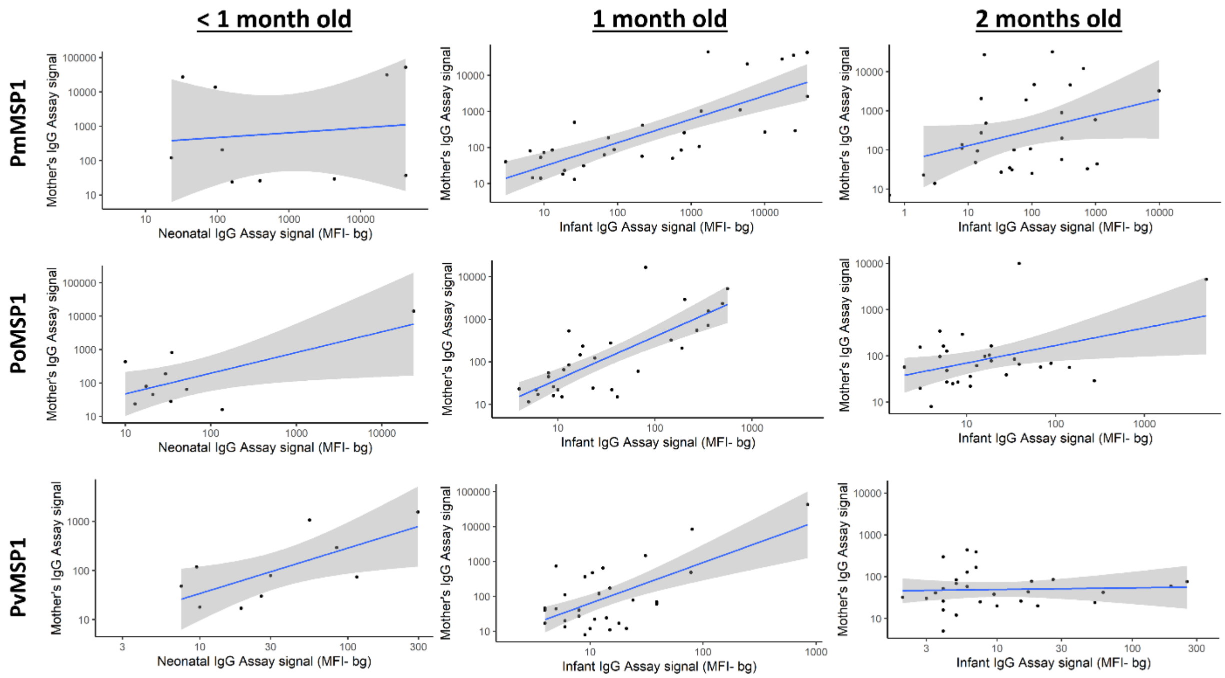

Supplementary Figure 4. Scatterplots of maternal and young infants' anti-malarial IgG levels against non-falciparum antigens. Solid line shows linear regression fitting for the data with shading indicating the 95% confidence interval.

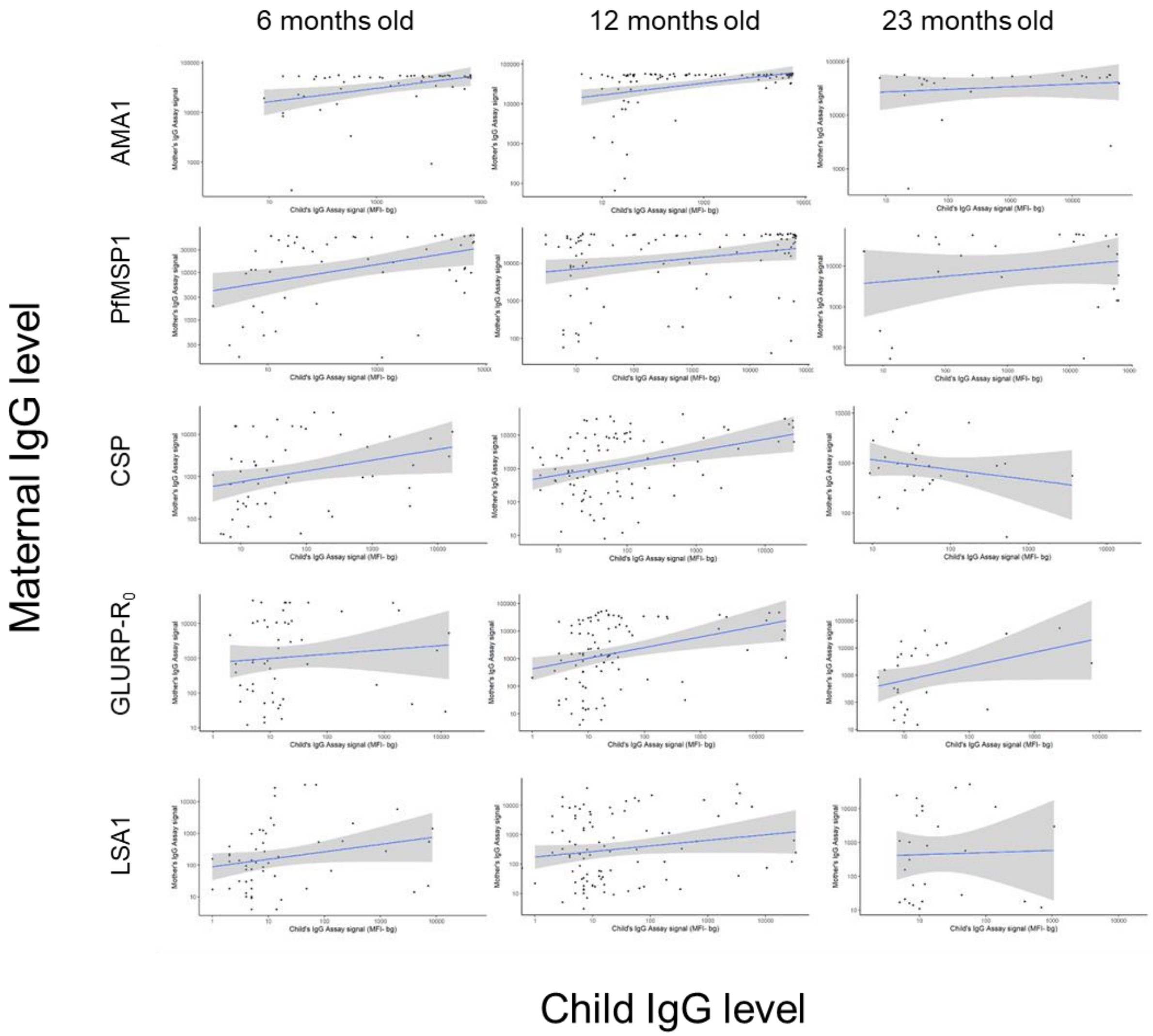

Supplementary Figure 5. Scatterplots of maternal and children's anti-malarial IgG levels against *P. falciparum* antigens for older infants and young children up to 2 years of age. Solid line shows linear regression fitting for the data with shading indicating the 95% confidence interval.

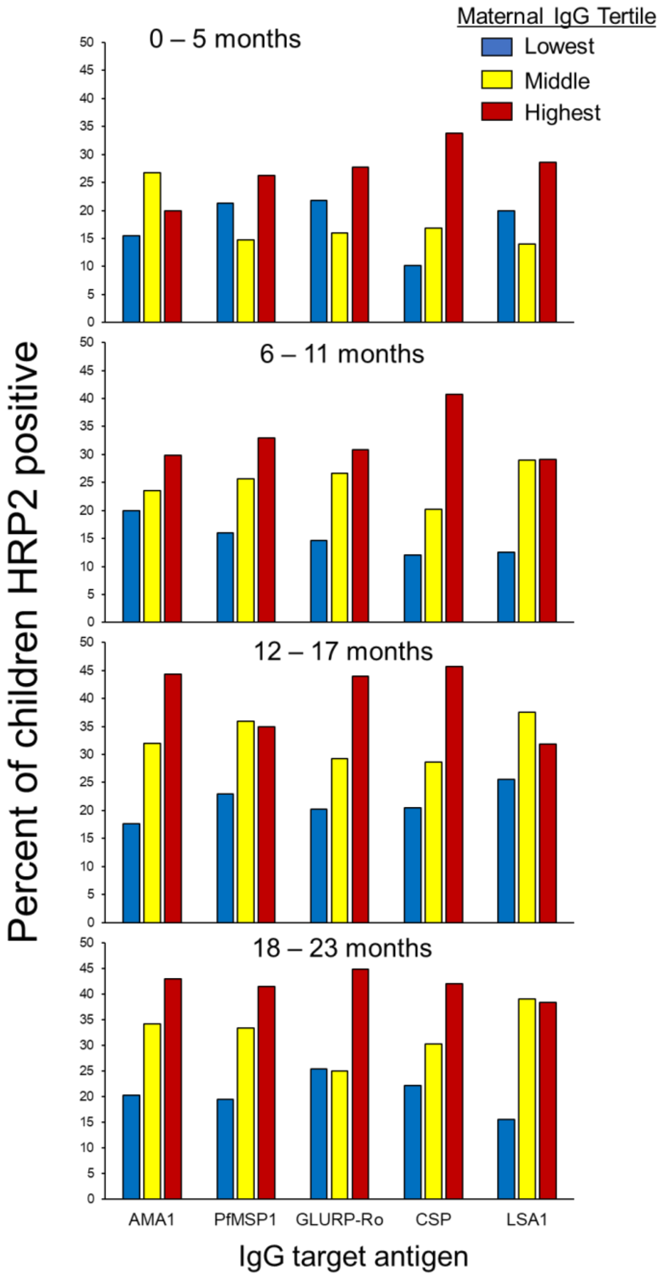

Supplementary Figure 6. Percentage of children positive for HRP2 antigen by maternal IgG levels for the five *P. falciparum* antigens. Plots separated by child age categories, and bar colors indicate maternal IgG tertile to each *P. falciparum* antigen. Each plot's y-axis is the percentage of children who are HRP2 antigen positive.
